# Association between unintentional injuries and suicidal ideation of children and adolescents: a longitudinal study

**DOI:** 10.3389/fpsyg.2026.1963201

**Published:** 2026-10-07

**Authors:** Yulian Yang, Biao Chen, Zhengyan Chen, Jingjing Lu, Xudong Zhou

**Affiliations:** 1 The Institute of Social and Family Medicine, School of Public Health, School of Medicine, Zhejiang University, Hangzhou, Zhejiang, China; 2 The Second Affiliated Hospital, Zhejiang University School of Medicine, Hangzhou, Zhejiang, China

**Keywords:** children and adolescents, longitudinal study, prospective association, suicidal ideation, unintentional injuries

## Abstract

**Purpose:**

This study aims to investigate the prospective association between unintentional injuries and subsequent suicidal ideation among children and adolescents.

**Methods:**

This study is a two-wave longitudinal school-based cohort derived from repeated surveys administered in Anhui, China, in November 2023 (Wave 1) and April 2025 (Wave 2). As grade 12 students in Wave 1 would graduate by July 2024 and thus be unavailable for follow-up, we included only students in grades 5–11 from Wave 1. We identified 24,522 students who participated in both waves, yielding a follow-up rate of 84.18% (47.08% female).

**Results:**

Among 12 types of unintentional injuries, after adjustment for socio-demographic characteristics and Wave 1 suicidal ideation, all 12 types were significantly associated with Wave 2 suicidal ideation, with adjusted odds ratios ranging from 1.31 to 1.65; all associations remained significant after Benjamini–Hochberg FDR correction (q < 0.05). The strongest associations were observed for explosion injuries (OR = 1.65, 95% CI = 1.27–2.13, q < 0.001), injuries from sharp objects (OR = 1.62, 95% CI = 1.50–1.75, q < 0.001), and poisoning (OR = 1.54, 95% CI = 1.19–1.99, q = 0.001), while the weakest, though still significant, was for Swallow a foreign object (OR = 1.31, 95% CI = 1.21–1.42, q < 0.001). Formal interaction tests indicated that only 3 of the 12 injury types (fall injuries, explosion injuries, and electric-current exposure) showed statistically significant gender × injury interactions, suggesting that the overall pattern of associations is largely consistent across sexes.

**Conclusion:**

Previous unintentional injuries are associated with an increased likelihood of subsequent suicidal ideation. Given the consistent pattern of associations and the absence of a clearly dominant injury category, broadly framed injury-prevention and early-identification strategies may be warranted, rather than prioritization of any single injury type.

## Introduction

Suicidal ideation, encompassing thoughts about the worthlessness of life and death wishes, represents a critical public health issue and a well-established risk factor for both psychiatric hospitalization and fatal suicidal behavior (Klonsky et al., 2016). Children and adolescents are particularly vulnerable to experiencing suicidal ideation because they navigate multiple physical, psychological, and social changes during adolescence (Bilsen, 2018). Globally, approximately 14% of adolescents report suicidal ideation (Biswas et al., 2020), with the highest pooled prevalence observed in Africa (21.0%) and the lowest in Asia (8.0%). Within China, about 16.3% of children and adolescents were at risk of engaging in suicidal ideation or had engaged in suicidal ideation in the previous year (Peng et al., 2021). Despite these alarming figures, the true prevalence is likely substantially higher due to underreporting, as many young individuals may conceal their psychological distress to avoid social stigma, labeling, or discrimination (Rüsch et al., 2014).

A growing body of research has explored risk factors associated with suicidal ideation in children and adolescents since suicidal ideation can be one of the major risk factors for suicidal death after a previous suicide attempt (Franklin et al., 2017; Goñi-Sarriés et al., 2018). A recent meta analysis (Chang et al., 2024) indicated that suicidal ideation was more prevalent among girls than boys, more common in middle and high school students compared to primary school students, and higher among adolescents residing in central China relative to other regions. Additional factor such as school bullying victimization (Van Geel et al., 2021), internet addiction (Peng et al., 2021), child–parent separation (Fellmeth et al., 2018), trauma and bereavement (Bilsen, 2018) are also associated with increased risk of suicidal ideation among children and adolescents. As noted, the COVID-19 pandemic has exacerbated this mental health crisis, with studies suggesting a 10% increase in the prevalence of suicidal ideation among adolescents in the post-pandemic period compared to pre-pandemic levels (Dubé et al., 2021). Therefore, more studies are needed for developing specific prevention strategies for children and adolescents’ suicidal ideation.

Further complicating the understanding and prevention of youth suicide is the fact that suicidal deaths can be misclassified as unintentional injuries—and vice versa—especially since suicidal ideation in young people is often underdetected and poorly documented (Freuchen et al., 2012). One previous study pointed out the association between caregiver-reported children’s unintentional injuries and self-reported deliberate self-harm behaviors in children (Lu et al., 2024), suggesting that some “accidental” injuries may represent intentional acts or undiagnosed suicidal behavior. Unintentional injuries are defined as injuries without purpose and consciousness and mainly include traffic injuries, falls, burns and scalds, poisoning, drowning, cutting injury and animal bites. According to the United Nations International Children’s Emergency Fund (UNICEF), injuries rank among the top three causes of death and lifelong disability among children aged 5–15 years, and approximately 1,600 children and adolescents die from unintentional injuries daily worldwide (United Nations International Children's Emergency Fund, 2025).

Generally speaking, the emergency department serves as the primary setting for initial data collection on both children and adolescents’ unintentional injuries and suicidal ideation and attempts. However, this source is inherently biased toward severe cases, as only those with serious injuries typically receive emergency department care, while less severe incidents may be treated in school infirmaries or at home. A study (Tong et al., 2019) of hotline callers in China concluded that many episodes of self-harm did not receive medical treatment, despite half of them reporting suicidal ideation, despite half of the individuals involved reporting concurrent suicidal ideation. Consequently, to more accurately elucidate the relationship between unintentional injuries and subsequent suicidal ideation, large-scale longitudinal studies are needed. Such designs can better capture underrepresented cases and allow for temporal sequencing between exposures and outcomes.

Based on these considerations, the current study aims to investigate the prospective association between unintentional injuries and subsequent suicidal ideation among children and adolescents, and to examine whether this relationship varies by gender. We propose the following hypotheses:

H1: Previous unintentional injuries are associated with later suicidal ideation among children and adolescents.

H2: The association between unintentional injuries and suicidal ideation varies across genders.

## Materials and methods

### Data collection

At the request of the NL County Education Department, we conducted large-scale mental health censuses biannually or annually since 2022. These censuses covered all students in grades 5–12 from 35 primary schools, 27 middle schools, and 6 high schools in NL County, Anhui Province. Detailed data collection procedures for these surveys are described in our prior publications (Lu et al., 2025). The present study utilized two waves of data: Wave 1 Collected in November 2023 with a response rate of 93.65%; Wave 2 Collected in April 2025 with a response rate of 94.56%.

As grade 12 students in Wave 1 would graduate by July 2024 and thus be unavailable for follow-up, we included only students in grades 5–11 from Wave 1 (29,129 of 33,086). Using name and birth date matching, we identified 24,522 students who participated in both waves, yielding a follow-up rate of 84.18%.

### Study variables

Students’ suicidal ideation (Wave 1 and Wave 2) was assessed by the last item of the Patient Health Questionnaire-9 (“thoughts that you would be better off dead or of hurting yourself in some way in the past 14 days”) (Kroenke et al., 2001). Responses of “not at all” were coded as “no suicidal ideation”; all others as “have suicidal ideation.”

Students’ 12 types of unintentional injuries sustained during the 2023 summer vacation were assessed separately with questions like “Have you ever suffered from vehicle and traffic injuries accidentally in the past summer vacation?”

Socio-demographic characteristics included gender, parental marital status, family economic status, paternal and maternal education in Wave 1, and age in Wave 2.

### Ethics

The ethics committee of our university reviewed and approved this study. Signed written consent was obtained from legal guardians of students. Additionally, we informed students that the survey was voluntary and obtained verbal consent from students before the commencement of the survey.

### Data analysis

Descriptive statistics, chi-square tests, and t-test were used to explore differences among students with and without suicidal ideation including unintentional injuries and socio-demographic characteristics. Then, we used logistic regression to examine the associations between suicidal ideation and unintentional injuries. The primary logistic model was adjusted for socio-demographic characteristics (age at Wave 2, gender, parental marital status, family economic status, and parental education) and Wave 1 suicidal ideation. Subgroup analyses stratified by gender were performed as descriptive summaries, and the gender × injury interaction was formally tested in the full sample by adding injury × gender interaction terms to the adjusted logistic model; interaction *p*-values are reported without multiplicity correction. To account for multiple testing across the 12 injury types, we applied the Benjamini–Hochberg false discovery rate (FDR) correction within the overall, male, and female analyses, and treated associations with FDR-adjusted q < 0.05 as statistically significant. All regression analyses used complete-case analysis. We analyzed all data using Python (version 3.13) with the statsmodels package; the main analyses were additionally cross-checked against the original SPSS output to confirm numerical consistency.

## Results

The descriptive statistics of the 24,522 students included in this study are summarized in Table 1. Females comprised approximately 47% of the sample, with a mean age of 14.71 years across all participants. 22.79% of students experienced parental divorce or other circumstances. Over 60% of participants indicated that at least one parent had attained education beyond primary school, and the majority perceived their family economic status as fair or wealthy.

**Table 1:** Descriptive statistics.

Variable	Total N(%)	Suicidal ideation in Wave2 N(%)		*p*-value
		No	Yes	
Gender				<0.001
Male	12,967(52.88)	10,880(55.88)	2087(41.32)	
Female	11,536(47.04)	8,577(44.05)	2,959(58.58)	
Missing	19(0.08)	14(0.07)	5(0.10)	
Age Mean(SD)	14.71(1.93)	14.73(1.93)	14.64(1.94)	0.001
Missing	0(0.00)	0(0.00)	0(0.00)	
Parental marital status				<0.001
Married	18,931(77.20)	15,331(78.74)	3,600(71.27)	
Divorced or other	5,588(22.79)	4,137(21.25)	1,451(28.73)	
Missing	3(0.01)	3(0.02)	0(0.00)	
Family economic status				<0.001
Poor	3,409(13.90)	2,522(12.95)	887(17.56)	
Fair	15,494(63.18)	12,432(63.85)	3,062(60.62)	
Wealthy	5,615(22.90)	4,514(23.18)	1,101(21.80)	
Missing	4(0.02)	3(0.02)	1(0.02)	
Paternal education				0.04
Primary school or below	3,032(12.36)	2,380(12.22)	652(12.91)	
Middle school	13,387(54.59)	10,713(55.02)	2,674(52.94)	
High school or above	6,414(26.16)	5,065(26.01)	1,349(26.71)	
Do not know	1,689(6.89)	1,313(6.74)	376(7.44)	
Missing	0(0.00)	0(0.00)	0(0.00)	
Maternal education				0.002
Primary school or below	4,152(16.93)	3,287(16.88)	865(17.13)	
Middle school	12,485(50.91)	10,025(51.49)	2,460(48.70)	
High school or above	5,976(24.37)	4,683(24.05)	1,293(25.60)	
Do not know	1909(7.78)	1,476(7.58)	433(8.57)	
Missing	0(0.00)	0(0.00)	0(0.00)	
Suicidal ideation in Wave 1				<0.001
No	19,403(79.12)	16,752(86.04)	2,651(52.48)	
Yes	5,115(20.86)	2,715(13.94)	2,400(47.52)	
Missing	4(0.02)	4(0.02)	0(0.00)	

A total of 36,015 cases of 12 types of unintentional injuries were reported by 24,522 students with the incidence of falls (39.05%) ranking the highest, followed by that of bitten or scratched by an animal (23.58%) and injuries from sharp objects (20.33%). Table 2 presents unintentional injuries of all 12 types by suicidal ideation. There were substantially more cases of unintentional injuries of all 12 types for students with suicidal ideation.

**Table 2:** Unintentional injuries of participants.

Variable N(%)	Total	Suicidal ideation in Wave2		p-value
		No	Yes	
Traffic injuries				<0.001
No	22,940(93.55)	18,367(94.33)	4,573(90.54)	
Yes	1,578(6.44)	1,100(5.65)	478(9.46)	
Missing	4(0.02)	4(0.02)	0(0.00)	
Fall injuries				<0.001
No	14,941(60.93)	12,463(64.01)	2,478(49.06)	
Yes	9,574(39.04)	7,001(35.96)	2,573(50.94)	
Missing	7(0.03)	7(0.04)	0(0.00)	
Hit by a falling object				<0.001
No	23,647(96.43)	18,905(97.09)	4,742(93.88)	
Yes	866(3.53)	560(2.88)	306(6.06)	
Missing	9(0.04)	6(0.03)	3(0.06)	
Collision or crush injuries from objects				<0.001
No	21,147(86.24)	17,152(88.09)	3,995(79.09)	
Yes	3,361(13.71)	2,308(11.85)	1,053(20.85)	
Missing	14(0.06)	11(0.06)	3(0.06)	
Explosion injuries				<0.001
No	24,189(98.64)	19,244(98.83)	4,945(97.90)	
Yes	316(1.29)	213(1.09)	103(2.04)	
Missing	17(0.07)	14(0.07)	3(0.06)	
Swallow a foreign object				<0.001
No	19,761(80.58)	16,093(82.65)	3,668(72.62)	
Yes	4,744(19.35)	3,365(17.28)	1,379(27.30)	
Missing	17(0.07)	13(0.07)	4(0.08)	
Bitten or scratched by an animal				<0.001
No	18,726(76.36)	15,328(78.72)	3,398(67.27)	
Yes	5,779(23.57)	4,133(21.23)	1,646(32.59)	
Missing	17(0.07)	10(0.05)	7(0.14)	
Drowning or near drowning incident				<0.001
No	24,004(97.89)	19,119(98.19)	4,885(96.71)	
Yes	502(2.05)	339(1.74)	163(3.23)	
Missing	16(0.07)	13(0.07)	3(0.06)	
Exposure to electric current				<0.001
No	23,710(96.69)	18,926(97.20)	4,784(94.71)	
Yes	794(3.24)	530(2.72)	264(5.23)	
Missing	18(0.07)	15(0.08)	3(0.06)	
Burns or scalds				<0.001
No	21,319(86.94)	17,269(88.69)	4,050(80.18)	
Yes	3,196(13.03)	2,196(11.28)	1,000(19.80)	
Missing	7(0.03)	6(0.03)	1(0.02)	
Poisoning				<0.001
No	24,188(98.64)	19,250(98.86)	4,938(97.76)	
Yes	321(1.31)	212(1.09)	109(2.16)	
Missing	13(0.05)	9(0.05)	4(0.08)	
Injuries from sharp objects				<0.001
No	19,530(79.64)	16,122(82.80)	3,408(67.47)	
Yes	4,984(20.32)	3,342(17.16)	1,642(32.51)	
Missing	8(0.03)	7(0.04)	1(0.02)	

### The associations between suicidal ideation and unintentional injuries

Table 3 presents regression coefficients for suicidal ideation on unintentional injuries adjusted for socio-demographic characteristics and Wave 1 suicidal ideation (24,489 complete cases included). Compared with students without suicidal ideation, all 12 unintentional injury types were positively associated with suicidal ideation after FDR correction. Adjusted odds ratios ranged from 1.31 (swallow a foreign object) to 1.65 (explosion injuries).

**Table 3:** Regression coefficients for unintentional injuries on suicidal ideation, adjusted for socio-demographic characteristics and Wave-1 suicidal ideation (overall sample, *n* = 24,489).

Variables	Suicidal ideation in Wave2		
	OR (95%CI)	p-value	q (FDR)
Traffic injuries	1.47 (1.30,1.66)	<0.001	<0.001
Fall injuries	1.48 (1.38,1.59)	<0.001	<0.001
Hit by a falling object	1.49 (1.27,1.74)	<0.001	<0.001
Collision or crush injuries from objects	1.44 (1.32,1.57)	<0.001	<0.001
Explosion injuries	1.65 (1.27,2.13)	<0.001	<0.001
Swallow a foreign object	1.31 (1.21,1.42)	<0.001	<0.001
Bitten or scratched by an animal	1.36 (1.26,1.46)	<0.001	<0.001
Drowning or near drowning incident	1.39 (1.13,1.71)	0.002	0.002
Exposure to electric current	1.44 (1.22,1.70)	<0.001	<0.001
Burns or scalds	1.46 (1.33,1.60)	<0.001	<0.001
Poisoning	1.54 (1.19,1.98)	0.001	0.001
Injuries from sharp objects	1.62 (1.50,1.75)	<0.001	<0.001
Gender			
Male	1.00		
Female	1.51(1.41,1.63)	<0.001	/
Age Mean(SD)	0.94(0.92,0.95)	<0.001	/
Parental marital status			
Married	1.00		
Divorced or other	1.14(1.05,1.25)	0.002	/
Family economic status			
Poor	1.00		
Fair	0.87(0.79,0.97)	0.009	/
Wealthy	0.87(0.77,0.98)	0.027	/
Paternal education			
Primary school or below	1.00		
Middle school	0.97(0.86,1.09)	0.608	/
High school or above	0.97(0.85,1.12)	0.710	/
Do not know	0.98(0.79,1.21)	0.830	/
Maternal education			
Primary school or below	1.00		
Middle school	0.98(0.88,1.09)	0.719	/
High school or above	1.02(0.90,1.16)	0.763	/
Do not know	1.05(0.86,1.29)	0.600	/
Suicidal ideation in Wave 1			
No	1.00		
Yes	3.88(3.58,4.21)	<0.001	/

### The gender difference

Among male students (12,956 complete cases included), 11 of the 12 unintentional injury types remained statistically significant after FDR correction, with the sole exception of explosion injuries (OR = 1.31, 95% CI = 0.94–1.84, q = 0.112) (Table 4). Adjusted odds ratios ranged from 1.33 to 1.76, with the largest effect observed for exposure to electric current (OR = 1.76, 95% CI = 1.43–2.18, q < 0.001).

**Table 4:** Regression coefficients for unintentional injuries on suicidal ideation, adjusted for socio-demographic characteristics and Wave-1 suicidal ideation (male students, n = 12,956).

Variables	Suicidal ideation in Wave2		
	OR(95%CI)	p-value	q (FDR)
Traffic injuries	1.33 (1.12, 1.57)	0.001	0.001
Fall injuries	1.37 (1.24, 1.51)	<0.001	<0.001
Hit by a falling object	1.61 (1.29, 2.00)	<0.001	<0.001
Collision or crush injuries from objects	1.54 (1.35, 1.75)	<0.001	<0.001
Explosion injuries	1.31 (0.94, 1.84)	0.112	0.112
Swallow a foreign object	1.35 (1.20, 1.52)	<0.001	<0.001
Bitten or scratched by an animal	1.40 (1.25, 1.57)	<0.001	<0.001
Drowning or near drowning incident	1.53 (1.16, 2.03)	0.003	0.003
Exposure to electric current	1.76 (1.42, 2.18)	<0.001	<0.001
Burns or scalds	1.50 (1.32, 1.70)	<0.001	<0.001
Poisoning	1.64 (1.17, 2.31)	0.004	0.004
Injuries from sharp objects	1.48 (1.32, 1.66)	<0.001	<0.001
Age Mean(SD)	1.04(1.01, 1.07)	0.015	/
Parental marital status			
Married	1.00		
Divorced or other	1.16(1.03, 1.32)	0.016	/
Family economic status			
Poor	1.00		
Fair	0.94(0.82, 1.09)	0.412	/
Wealthy	0.90(0.76, 1.07)	0.253	/
Paternal education			
Primary school or below	1.00		
Middle school	0.96(0.81, 1.14)	0.644	/
High school or above	1.02(0.84, 1.25)	0.815	/
Do not know	0.84(0.62, 1.15)	0.286	/
Maternal education			
Primary school or below	1.00		
Middle school	0.92(0.79, 1.07)	0.256	/
High school or above	0.97(0.81, 1.17)	0.743	/
Do not know	1.05(0.79, 1.39)	0.749	/
Suicidal ideation in Wave 1			
No	1.00		
Yes	4.00(3.54, 4.53)	<0.001	/

Among female students (11,530 complete cases included), 9 of the 12 unintentional injury types remained statistically significant after FDR correction; the three non-significant types were drowning or near-drowning (OR = 1.26, 95% CI = 0.92–1.72, q = 0.166), exposure to electric current (OR = 1.12, 95% CI = 0.86–1.46, q = 0.388), and poisoning (OR = 1.38, 95% CI = 0.93–2.06, q = 0.129) (Table 5). Adjusted odds ratios ranged from 1.12 to 2.45, with explosion injuries showing the largest effect (OR = 2.45, 95% CI = 1.59–3.76, q < 0.001).

**Table 5:** Regression coefficients for unintentional injuries on suicidal ideation, adjusted for socio-demographic characteristics and Wave-1 suicidal ideation (female students, *n* = 11,530).

Variables	Suicidal ideation in Wave2		
	OR(95%CI)	p-value	q (FDR)
Traffic injuries	1.67 (1.39, 2.00)	<0.001	<0.001
Fall injuries	1.60 (1.46, 1.75)	<0.001	<0.001
Hit by a falling object	1.40 (1.11, 1.76)	0.004	0.006
Collision or crush injuries from objects	1.39 (1.23, 1.57)	<0.001	<0.001
Explosion injuries	2.45 (1.59, 3.76)	<0.001	<0.001
Swallow a foreign object	1.31 (1.18, 1.46)	<0.001	<0.001
Bitten or scratched by an animal	1.38 (1.25, 1.52)	<0.001	<0.001
Drowning or near drowning incident	1.26 (0.92, 1.72)	0.152	0.166
Exposure to electric current	1.12 (0.86, 1.46)	0.388	0.388
Burns or scalds	1.47 (1.29, 1.67)	<0.001	<0.001
Poisoning	1.38 (0.93, 2.06)	0.107	0.129
Injuries from sharp objects	1.76 (1.59, 1.96)	<0.001	<0.001
Age Mean(SD)	0.86(0.83, 0.88)	<0.001	/
Parental marital status			
Married	1.00		
Divorced or other	1.11(0.99, 1.25)	0.073	/
Family economic status			
Poor	1.00		
Fair	0.82(0.71, 0.95)	0.009	/
Wealthy	0.82(0.69, 0.98)	0.026	/
Paternal education			
Primary school or below	1.00		
Middle school	0.97(0.83, 1.14)	0.746	/
High school or above	0.92(0.76, 1.11)	0.395	/
Do not know	1.07(0.80, 1.45)	0.638	/
Maternal education			
Primary school or below	1.00		
Middle school	1.03(0.89, 1.19)	0.908	/
High school or above	1.06(0.89, 1.27)	0.649	/
Do not know	1.08(0.81, 1.44)	0.494	/
Suicidal ideation in Wave1			
No	1.00		
Yes	3.85(3.44, 4.30)	<0.001	/

Formal tests of the gender × injury interaction showed statistically significant interactions for only 3 of the 12 injury types (fall injuries, explosion injuries, and exposure to electric current; Table 6), indicating that the overall pattern of associations was largely consistent across sexes.

**Table 6:** Tests of gender × injury interaction for the associations between unintentional injuries and suicidal ideation in Wave 2.

Injury type	Interaction OR (95% CI)	p for interaction
Traffic injuries	1.19 (0.93, 1.52)	0.164
Fall injuries	1.27 (1.11, 1.45)	<0.001
Hit by a falling object	0.93 (0.68, 1.28)	0.672
Collision or crush injuries from objects	0.84 (0.71, 1.00)	0.051
Explosion injuries	2.03 (1.18, 3.49)	0.011
Swallow a foreign object	1.01 (0.86, 1.18)	0.935
Bitten or scratched by an animal	0.92 (0.80, 1.07)	0.293
Drowning or near drowning incident	0.89 (0.58, 1.35)	0.574
Exposure to electric current	0.69 (0.49, 0.96)	0.029
Burns or scalds	1.02 (0.85, 1.22)	0.826
Poisoning	0.83 (0.49, 1.39)	0.469
Injuries from sharp objects	1.16 (1.00, 1.35)	0.051

Each row presents the injury × gender interaction term from a separate multivariable logistic regression model predicting suicidal ideation in Wave 2, adjusted for the injury main effect, gender, age, parental marital status, family economic status, paternal and maternal education, and Wave1 suicidal ideation. *p* values are from two-sided Wald tests and were not adjusted for multiple comparisons, as the interaction tests were a secondary analysis and were not included in the Benjamini–Hochberg correction applied to the primary associations (Tables 3–5). The significant interaction for explosion injuries is based on few exposed participants and should be interpreted with caution.

Table 7 compares the baseline characteristics between participants who were followed up and those who were lost to follow-up. No significant gender difference was observed between the two groups (*p* = 0.180). Compared with followed participants, non-followed participants were more likely to have parents with divorced or other marital status (30.83% vs. 22.79%), to perceive their family economic status as poor (18.99% vs. 13.90%), and to have lower paternal and maternal educational attainment. They also had a higher prevalence of suicidal ideation (29.04% vs. 20.86%) at Wave 1.

**Table 7:** Baseline characteristics between followed and non-followed participants.

Variable	Followed		*p*-value
	No	Yes	
Gender			0.180
Male	2,499(54.24)	12,967(52.88)	
Female	2,103(45.65)	11,536(47.04)	
Parental marital status			<0.001
Married	3,186(69.17)	18,931(77.21)	
Divorced or other	1,420(30.83)	5,588(22.79)	
Family economic status			<0.001
Poor	875(18.99)	3,409(13.90)	
Fair	2,798(60.73)	15,494(63.19)	
Wealthy	934(20.27)	5,615(22.90)	
Paternal education			<0.001
Primary school or below	734(15.93)	3,032(12.36)	
Middle school	2,445(53.07)	13,387(54.59)	
High school or above	1,060(23.01)	6,414(26.16)	
Do not know	368(7.99)	1,689(6.89)	
Maternal education			<0.001
Primary school or below	936(20.32)	4,152(16.93)	
Middle school	2,263(49.12)	12,485(50.91)	
High school or above	962(20.88)	5,976(24.37)	
Do not know	446(9.68)	1909(7.78)	
Suicidal ideation in Wave 1			<0.001
No	3,269(70.96)	19,403(79.14)	
Yes	1,338(29.04)	5,115(20.86)	

## Discussion

To the best of our knowledge, this is the first study to use a large-scale county-wide school-based longitudinal design to examine the associations between different types of unintentional injuries and suicidal ideation among children and adolescents in China. We found that among 12 types of unintentional injuries, all 12 types were positively associated with Wave 2 suicidal ideation after adjustment for socio-demographic characteristics and Wave 1 suicidal ideation, and all 12 associations remained statistically significant after Benjamini–Hochberg FDR correction. The magnitudes of the adjusted associations were modest but consistent (odds ratios of approximately 1.31–1.65), indicating that unintentional injuries are statistically significant but modest correlates of suicidal ideation and are likely to represent only one of many contributing factors. The relatively narrow range of effect sizes across the 12 injury types further suggests that, at the population level, the category-specific contributions to suicidal-ideation risk are broadly similar.

Several, not mutually exclusive, mechanisms may account for these associations. First, unintentional injuries can be conceptualized as stressful and, in some cases, potentially traumatic events. Injuries that involve pain, medical treatment, activity restriction, or absence from school can generate considerable psychological distress during a developmental period in which coping resources are still maturing, and psychological distress and hopelessness are proximal correlates of suicidal ideation in young people (Bilsen, 2018; Klonsky et al., 2016). Second, injuries often carry interpersonal and social consequences: they can disrupt peer relationships and school connectedness, restrict participation in age-typical activities, and impose caregiving or financial demands on families, all of which may foster perceived burdensomeness and thwarted belongingness—antecedents of suicidal ideation emphasized by the interpersonal theory of suicide (Van Orden et al., 2010). Third, part of the association may reflect unmeasured vulnerability rather than a causal effect of the injury itself: children who sustain injuries may also engage in risk-taking behavior or experience family or social adversities that independently increase the likelihood of later suicidal ideation. In addition, some injuries reported as unintentional may actually represent unreported deliberate self-harm or risky behavior arising from pre-existing distress (Freuchen et al., 2012; Lu et al., 2024), which could also contribute to the observed associations. We emphasize, however, that the present observational design does not permit causal inference, and that none of these mechanisms could be directly tested with the available data; our findings therefore support an association between unintentional injuries and subsequent suicidal ideation rather than a demonstrated causal pathway.

The relatively consistent pattern of associations between exposure to unintentional injuries and subsequent suicidal ideation observed in both male and female students warrants attention. Although the modest magnitude of the estimates indicates that unintentional injuries as a whole are best regarded as one of several markers of vulnerability rather than a strong determinant, the consistency of the associations across the 12 injury categories (all FDR-significant in the full sample) suggests that injury exposure may be acting as a general, non-specific indicator of psychological distress in this age group. The absence of a single dominant injury category in the adjusted models reinforces this interpretation. This pattern is consistent with the broader literature linking painful or injurious experiences to later suicide-related outcomes in young people. Related work on non-suicidal self-injury and adverse childhood experiences has similarly highlighted the role of accumulated physical and psychological stressors in elevating later suicide risk, although the present findings are best interpreted in the context of a stress-distress framework rather than a capability-based one, given that the outcome under study is ideation rather than behavior.

The current study offers several important contributions to the existing literature. First, through a large-scale county-wide school-based survey conducted in NL County with high response and follow-up rates, the study minimizes potential sampling biases, thereby enhancing the representativeness and generalizability of the findings. Second, by examining 12 specific types of unintentional injuries separately rather than as a composite measure, the study provides a fine-grained picture of which injury categories carry the strongest association with subsequent suicidal ideation. Third, although stratified estimates suggested somewhat larger effects among female students for some injury types (notably fall and explosion injuries), formal interaction tests did not support a consistent gender-specific pattern across the 12 injury categories (Table 6). Therefore, the gender-stratified results should be interpreted as descriptive rather than as evidence of effect modification, and the present data do not justify gender-specific allocation of intervention resources. Taken together, the present study reinforces the public-health relevance of unintentional injuries as a class of risk markers for suicidal ideation among children and adolescents.

The current study has several limitations that should be considered when interpreting the findings. First, although the sample size was substantial and the follow-up rate was high, the research was conducted solely within a single county in China and included only students in grades 5–11. This may limit the generalizability of the findings to other regions, age groups, or to out-of-school youth. Second, although we adjusted for socio-demographic characteristics and Wave 1 suicidal ideation in the primary model, residual confounding from unmeasured factors (e.g., substance use, sleep disturbance, prior mental-health diagnoses, family history of suicidal behavior) is possible, and thus our findings should be interpreted as associative rather than causal. Moreover, the psychological mechanisms hypothesized to link unintentional injuries to suicidal ideation (e.g., perceived burdensomeness, reduced school connectedness, post-injury depressive symptoms) were not directly measured in the present study, so these explanatory pathways could not be empirically examined and remain speculative.

## Conclusion

Our findings underscore the public health relevance of unintentional injuries as a class of potential risk factors for suicidal ideation among children and adolescents. The largely comparable effect sizes across the 12 injury types suggest that broadly framed injury-prevention strategies may be more appropriate than narrow focus on any single category. Future research might employ experimental designs to evaluate whether interventions targeting post-injury psychological support can reduce the subsequent risk of suicidal ideation in this population.

## Data Availability

The raw data supporting the conclusions of this article will be made available by the authors, without undue reservation.
